# Current Strategies and Therapeutic Applications of Mesenchymal Stem Cell-Based Drug Delivery

**DOI:** 10.3390/ph17060707

**Published:** 2024-05-30

**Authors:** Yasunari Matsuzaka, Ryu Yashiro

**Affiliations:** 1 Division of Molecular and Medical Genetics, Center for Gene and Cell Therapy, The Institute of Medical Science, The University of Tokyo, Minato-ku, Tokyo 108-8639, Japan; 2 Administrative Section of Radiation Protection, National Institute of Neuroscience, National Center of Neurology and Psychiatry, Kodaira 187-8551, Tokyo, Japan; ryuy@niid.go.jp; 3 Department of Medical Molecular Informatics, Meiji Pharmaceutical University, Kiyose 204-8588, Tokyo, Japan; 4 Department of Mycobacteriology, Leprosy Research Center, National Institute of Infectious Diseases, Tokyo 162-8640, Japan

**Keywords:** mesenchymal stem cell, stem cell membrane-coated nanoparticles, stem cell-derived extracellular vesicles, immunomodulatory, stem cell-laden scaffolds, scaffold-free stem cell sheets

## Abstract

Mesenchymal stem cells (MSCs) have emerged as a promising approach for drug delivery strategies because of their unique properties. These strategies include stem cell membrane-coated nanoparticles, stem cell-derived extracellular vesicles, immunomodulatory effects, stem cell-laden scaffolds, and scaffold-free stem cell sheets. MSCs offer advantages such as low immunogenicity, homing ability, and tumor tropism, making them ideal for targeted drug delivery systems. Stem cell-derived extracellular vesicles have gained attention for their immune properties and tumor-homing abilities, presenting a potential solution for drug delivery challenges. The relationship between MSC-based drug delivery and the self-renewal and differentiation capabilities of MSCs lies in the potential of engineered MSCs to serve as effective carriers for therapeutic agents while maintaining their intrinsic properties. MSCs exhibit potent immunosuppressive functions in MSC-based drug delivery strategies. Stem cell-derived EVs have low immunogenicity and strong therapeutic potential for tissue repair and regeneration. Scaffold-free stem cell sheets represent a cutting-edge approach in regenerative medicine, offering a versatile platform for tissue engineering and regeneration across different medical specialties. MSCs have shown great potential for clinical applications in regenerative medicine because of their ability to differentiate into various cell types, secrete bioactive factors, and modulate immune responses. Researchers are exploring these innovative approaches to enhance drug delivery efficiency and effectiveness in treating various diseases.

## 1. Introduction

Mesenchymal stem cell (MSC)-based drug delivery strategies play a crucial role in biomedical applications, particularly in the field of skin regeneration and wound healing [1,2,3]. These strategies involve utilizing stem cells as carriers for drug delivery to enhance tissue regeneration and wound repair. Stem cells, due to their self-renewal and differentiation capabilities, offer promising potential for accelerating wound closure, skin regeneration, and preventing wound complications [4,5]. Various types of stem cells, such as embryonic stem cells (ESCs), adult stem cells (ASCs), MSCs, and induced pluripotent stem cells (iPSCs), have been explored for their roles in wound repair. Stem cell-based drug delivery systems include innovative approaches such as stem cell membrane-coated nanoparticles (SCMNPs), stem cell-derived extracellular vesicles (EVs), stem cell-laden scaffolds, and scaffold-free stem cell sheets [6]. These strategies hold significant promise as novel approaches for skin regeneration and wound healing by addressing challenges such as optimal source selection, processing methods, administration techniques, and cell survival at wound sites [1,7,8]. These strategies leverage the self-renewal and differentiation capabilities of MSCs to accelerate wound closure, promote skin regeneration, and prevent scarring. Various methods such as SCMNPs, stem cell-derived EVs, stem cells as drug carriers, scaffold-free stem cell sheets, and stem cell-laden scaffolds have been investigated in this field to enhance the effectiveness of drug delivery for optimal outcomes in wound repair [1,9].

MSCs, known for their self-renewal capacity and ability to differentiate into multiple cell lineages, have shown significant potential in cell therapy for a wide range of conditions. These include dermatological, musculoskeletal, neurological, cardiovascular, respiratory, renal, gastroenterological, and urological disorders [10]. In clinical trials, MSCs have demonstrated anti-fibrotic, anti-apoptotic, anti-inflammatory, immunomodulatory, and immunosuppressive effects, making them valuable for tissue regeneration in non-autologous cell therapy applications [10]. Studies have highlighted the efficacy of MSCs in treating conditions such as multiple sclerosis (MS), where intravenous injections of autologous BM-MSCs have shown safety and efficacy in patients [11]. Additionally, other types of MSCs, such as human placental MSCs and umbilical cord MSCs, have displayed beneficial effects in clinical studies for conditions such as MS and other neurodegenerative diseases [11].

However, MSCs are not inherently immunosuppressive but require specific activation or priming to display their full immunosuppressive capacities. Several factors can enhance the immunosuppressive properties of MSCs. Exposing MSCs to hypoxic conditions can upregulate the expression of immunosuppressive factors such as indoleamine 2,3-dioxygenase (IDO), prostaglandin E2 (PGE2), and transforming growth factor-beta (TGF-β), enhancing their ability to suppress T cell proliferation and activation [12]. Priming MSCs with inflammatory cytokines such as interferon-gamma (IFN-γ) and tumor necrosis factor-alpha (TNF-α) can increase their expression of immunomodulatory molecules such as programmed death-ligand 1 (PD-L1) and induce the secretion of anti-inflammatory factors such as interleukin-10 (IL-10). Certain drugs, such as dexamethasone, can also enhance the immunosuppressive properties of MSCs by upregulating the expression of immunomodulatory molecules [12,13]. Culturing MSCs in a 3D environment, such as hydrogels or spheroids, can maintain their immunosuppressive phenotype better than traditional 2D culture conditions. While MSCs possess inherent immunomodulatory capabilities, their full immunosuppressive potential is only realized when they are appropriately activated or primed, for example, through exposure to hypoxia, pro-inflammatory cytokines, pharmacological agents, or 3D culture conditions. These activation strategies can enhance the expression of key immunosuppressive factors and molecules, thereby improving the therapeutic efficacy of MSCs in various autoimmune and inflammatory conditions [12,13].

MSCs and their secreted EVs have shown great potential as delivery vehicles for therapeutic agents in tissue regeneration and cancer treatment. The principal methods for loading cargo into MSCs and EVs can be broadly classified into two categories: cell-based loading and non-cell-based loading. In cell-based loading, the cargo is first delivered to the donor MSCs, which is then packaged and secreted with the cargo within EVs. The transfection method involves using transfection reagents to introduce biomolecules such as small RNAs, mRNAs, DNAs, and proteins into MSCs. The cargo is then loaded into EVs secreted by the transfected MSCs. It allows the loading of large cargo with relatively high efficiency but requires transfection reagents and relies on transfection efficiency [14]. In passive incubation, small molecule drugs such as doxorubicin and curcumin can be passively loaded into MSCs by simple incubation. The drugs are then incorporated into EVs secreted by the MSCs. This method is simple but has low loading efficiency and may affect EV components [14].

As for non-cell-based loading methods, these methods involve directly loading cargo into isolated EVs without using donor cells. The electroporation, sonication, and freeze–thaw techniques can load small molecules and biomolecules such as RNAs, mRNAs, DNAs, and proteins into EVs. They have relatively high loading efficiency but may increase EV instability, cause aggregation, and require additional equipment or washing steps [14]. In passive incubation, similar to cell-based passive loading, small molecule drugs can be directly incubated with isolated EVs for encapsulation. While very simple, it suffers from low loading efficiency [14]. Regarding specific cargo, doxorubicin (an anticancer drug) has been successfully loaded into both MSC-derived EVs using passive incubation and active methods such as electroporation and sonication [15]. The loading efficiencies varied based on the source of EVs and the loading method used. Therefore, MSCs and their EVs can be engineered as delivery vehicles for various therapeutic agents using cell-based or non-cell-based cargo loading approaches, each with its own advantages and limitations in terms of cargo types, loading efficiency, and potential impact on EV integrity.

Engineered MSCs are indeed designed to enhance their therapeutic efficacy by increasing their survival in vivo through genetic modifications. This is supported by the following key points from the search results. The genetic modification of MSCs aims to improve their therapeutic potential by enhancing their survival, proliferation, migration, homing ability, and pro-regenerative capacity [16]. Overexpressing certain genes can increase MSC survival and proliferation rates [16,17]. Poor survival rate and proliferation of transplanted MSCs have been major limitations, possibly due to the hostile microenvironment of lesioned tissues [16]. Genetic engineering approaches have been explored to overcome this challenge [18,19]. Specific genetic modifications studied include overexpressing anti-apoptotic genes, growth factors, cytokines, and other factors that promote MSC survival, proliferation, and migration to injury sites [16,17,18,19]. In preclinical models of acute respiratory distress syndrome (ARDS), genetically engineered MSCs overexpressing therapeutic factors have shown improved ability to recover lung function, reduce inflammation, and enhance therapeutic effects compared with unmodified MSCs [19,20]. Thus, increasing the in vivo survival and therapeutic potency of MSCs through genetic engineering is a key strategy highlighted across multiple sources to overcome the limitations of conventional MSC therapy [16,17,18,19,20].

Furthermore, the secretory functions of MSCs play a crucial role in their therapeutic effects. The paracrine actions of MSCs through growth factors, cytokines, chemokines, and other secretory factors contribute to immune modulation, tissue remodeling, and cellular homeostasis during regeneration [21,22,23]. This paracrine function is essential for the immunosuppressive functions of MSCs and has been linked to their therapeutic efficacy in various inflammatory diseases [21]. Thus, the self-renewal and differentiation capabilities of MSCs offer a promising avenue for regenerative medicine and cell-based therapies across a spectrum of medical conditions. Ongoing research continues to explore the full potential of MSCs in treating diseases and injuries through their regenerative properties and secretory functions. In this review, we summarize current MSC-based drug delivery and therapeutic applications.

## 2. The Self-Renewal and Differentiation Capabilities of MSCs in MSC-Based Drug Delivery

### 2.1. Self-Renewal and Differentiation Capabilities of MSCs

**Figure 1:**
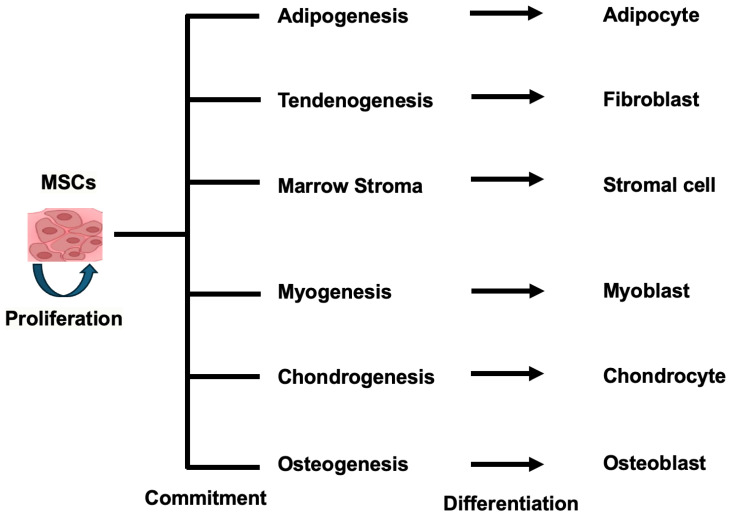
The multipotency allows MSCs to differentiate into different mesenchymal tissues.

MSCs possess two key features that define them as true stem cells: self-renewal and multilineage differentiation potential. MSCs can self-renew, meaning they can divide and maintain their undifferentiated state over extended periods of time. This self-renewal capacity allows MSCs to replenish the stem cell pool and maintain their stem cell properties [24]. In addition, MSCs exhibit multilineage differentiation potential, meaning they can give rise to various cell types of the mesenchymal lineage, including osteoblasts (bone cells), chondrocytes (cartilage cells), adipocytes (fat cells), and myocytes (muscle cells). This multipotency allows MSCs to differentiate and contribute to the regeneration of different mesenchymal tissues [25,26] (Figure 1). The self-renewal and multilineage differentiation capabilities of MSCs make them a valuable cell type for regenerative medicine and tissue engineering applications. Ongoing research aims to elucidate further the molecular mechanisms regulating MSC self-renewal and lineage-specific differentiation to harness their full therapeutic potential. 

MSCs are multipotent progenitor cells that can differentiate into various cell lineages, including adipocytes, chondrocytes, and osteoblasts, under defined in vitro conditions [27]. However, the in vivo differentiation capabilities of MSCs are less clear and require further investigation. In vitro, MSCs can be induced to differentiate into different lineages by culturing them on substrates with specific compliance, curvature, and dynamic movement, which may mimic the in vivo microenvironment. Additionally, the growth of MSCs on soft materials may help preserve their regenerative properties and allow them to recover from tissue culture-related aging. The in vivo identity and biology of MSCs are less well characterized compared with their in vitro behavior [25]. While MSCs have been shown to contribute to the repair of bones, muscles, tendons, and cartilage in various animal models, the extent to which they directly differentiate into these tissues versus exerting paracrine effects is still being investigated. Some studies have suggested that the tissue dissociation, adhesion to tissue culture plastic, and growth in serum-supplemented medium used to isolate and expand MSCs in vitro may produce a “valuable artifact” that does not necessarily reflect their true in vivo identity and differentiation potential [28]. The in vivo equivalents of in vitro-expanded MSCs remain obscure, and further research is needed to understand their biological properties in their native environments [29]. While MSCs demonstrate remarkable multipotency in vitro, their in vivo differentiation capabilities and implications for regenerative therapies require more careful examination and validation through additional in vivo studies.

The self-renewal and differentiation capabilities of MSCs play a crucial role in MSC-based drug delivery. MSCs are adult stem cells known for their ability to self-renew and differentiate into various cell lineages, making them valuable for regenerative medicine and drug delivery applications [2,29,30]. These capabilities allow MSCs to be engineered to enhance their survival, retention, migration, and growth factor production, which are essential for effective drug delivery [7]. Additionally, MSCs can be loaded with therapeutic agents and used for tissue regeneration and cancer therapeutics without compromising their differentiation potential [2,30]. The use of genetic modifications and engineering approaches can further improve the in vivo performance of MSCs in drug delivery applications [30]. Overall, the unique properties of MSCs make them promising candidates for efficient drug delivery systems in regenerative medicine and other therapeutic areas [31,32].

The relationship between MSC-based drug delivery and the self-renewal and differentiation capabilities of MSCs lies in the potential of engineered MSCs to serve as effective carriers for therapeutic agents while maintaining their intrinsic properties. MSCs, known for their self-renewal and differentiation abilities, are being engineered to enhance their survival, retention, migration, and growth factor production for regenerative medicine and drug delivery purposes [2,18,33]. Engineered MSCs are designed to improve their therapeutic efficacy by increasing their survival in vivo through genetic modifications that enhance pro-survival, pro-angiogenic, or anti-apoptotic gene expression. These modifications aim to address challenges such as poor survival, retention, and engraftment of MSCs in vivo, thus boosting their regenerative potential [22,33]. 

When used for drug delivery purposes, MSCs can be loaded with therapeutic agents such as protein-based drugs to treat conditions including cancer and genetic diseases. This approach involves modifying MSCs to produce protein-based therapeutics exogenously. Additionally, MSCs can be loaded with small therapeutic cargo directly without carrier encapsulation, showcasing their versatility as drug delivery vehicles [2,18,22]. Therefore, the relationship between MSC-based drug delivery and the self-renewal and differentiation capabilities of MSCs is that engineered MSCs can maintain their regenerative properties while being utilized as effective carriers for therapeutic agents. This dual functionality highlights the versatility and potential of MSCs in regenerative medicine and targeted drug delivery applications.

### 2.2. Developing Methods to Study the Differentiation of Human Mesenchymal Stem Cells

The research articles provided focus on developing methods to study the differentiation of human mesenchymal stem cells (hMSCs) under various conditions. These studies aim to understand how hMSCs can be differentiated using cyclic compressive strain alone, control MSC adhesion and differentiation in vitro, transform MSCs into hepatocyte-like cells, and analyze the aging process of MSCs and its impact on immunomodulatory activity. The studies explore different approaches, such as applying cyclic compressive strain, using silane-modified surfaces, employing transcription regulation and chromatin remodeling, and conducting multi-omics analysis to investigate the differentiation potential of hMSCs [34]. These approaches involve techniques such as adenosine triphosphate assays, live–dead cell assays, quantitative real-time polymerase chain reaction, single-cell transcriptomic analysis, and proteomic data integration [35,36,37,38]. 

Therefore, these studies contribute to advancing our understanding of how physical stimulation and biochemical cues influence the differentiation of hMSCs, paving the way for potential applications in tissue engineering, regenerative medicine, and biotechnology.

## 3. The Immunosuppressive Functions of MSCs

### 3.1. Immunosuppressive Functions of MSCs

The immunosuppressive functions of MSCs play a crucial role in MSC-based drug delivery strategies. MSCs have been engineered to express specific immunomodulatory agents, enhancing their immunomodulatory capacity and enabling them to deliver large doses of cancer-targeting biologics with a single dose [34]. These engineered MSCs can express diverse immunomodulatory molecules such as interferons (IFNs) and interleukins (ILs), contributing to their immunosuppressive functions [34]. Additionally, the immunosuppressive properties of MSCs are activated by immune-related factors such as interferon-gamma (IFN-γ) and pro-inflammatory cytokines such as TNF-α, IL-1α, and IL-1β, which are essential for their therapeutic effects in MSC-based drug delivery strategies [39]. The ability of MSCs to modulate the activity of surrounding cells through complex pathways involving various factors and signaling pathways further highlights their significance in immunosuppression for effective drug delivery strategies [39].

These functions play a crucial role in the therapeutic effects of MSCs. Some examples of the immunosuppressive functions of MSCs in drug delivery include the inhibition of immune responses: MSCs can inhibit immune responses mediated by active lymphocytes in a dose-dependent manner, showcasing their immunosuppressive capabilities [21]. In addition, promotion of orthopedic repair and wound healing: Allogenic MSCs have been shown to promote orthopedic repair, skin wound healing, and nerve regeneration/reconnection, highlighting their role in tissue repair and regeneration [21]. Also, numerous clinical trials have been registered to explore the applications of MSCs in inflammation, wound healing, infection, organ dysfunction, and degenerative diseases across various organs and tissues, emphasizing the broad therapeutic potential of MSC-based strategies [21]. These examples illustrate how MSCs possess immunosuppressive properties that are harnessed in drug-delivery approaches for therapeutic purposes.

**Figure 2:**
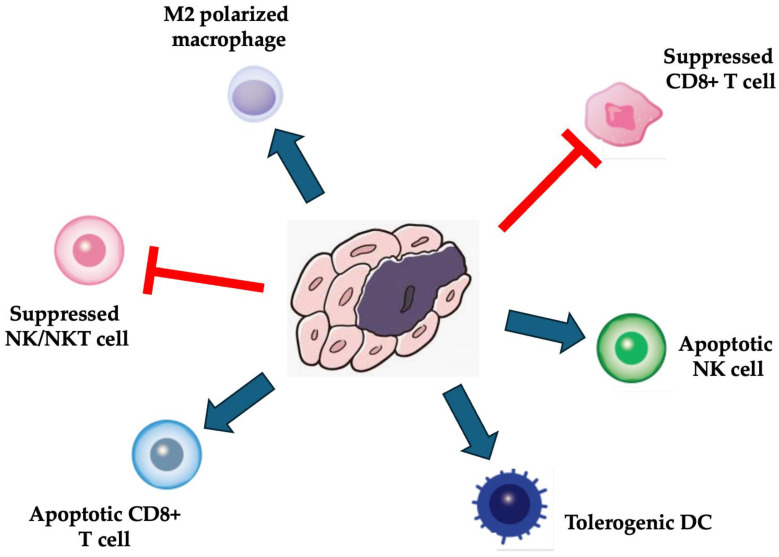
Immunosuppressive functions of MSCs.

Based on the provided sources, MSCs indeed demonstrate potent immunosuppressive functions in various diseases. These functions are attributable to the interactions of MSCs with immune cells, leading to immunomodulation and inhibition of immune responses. MSCs have been extensively studied for their ability to modulate the immune system by interacting with different immune cell types, such as T cells, B cells, natural killer cells, macrophages, dendritic cells, and neutrophils (Figure 2) [21,22,40]. They inhibit T-cell proliferation and function, regulate the maturation of dendritic cells, and interact with regulatory T-cells and monocytes to exert immunomodulatory effects [21]. Additionally, MSCs can inhibit immune responses by releasing soluble factors and suppressing the proliferation of various immune cells [22]. These findings support the statement that MSCs possess significant immunosuppressive properties across a range of diseases.

MSCs are not inherently immunogenic and can create an immunosuppressive environment through several mechanisms. MSCs lack the expression of costimulatory molecules such as CD80 and CD86, which are required to activate T cells fully [41]. They also have low expression of MHC class II molecules, further reducing their ability to trigger an immune response. This makes MSCs poorly immunogenic and less likely to be rejected when administered to an allogeneic recipient [42]. MSCs can actively suppress the immune system through the secretion of various soluble factors. They produce immunosuppressive molecules such as indoleamine 2,3-dioxygenase (IDO), prostaglandin E2 (PGE2), transforming growth factor-β (TGF-β), and hepatocyte growth factor (HGF) [41]. These factors inhibit the proliferation and activation of T cells, B cells, natural killer cells, and dendritic cells. MSCs can also induce the differentiation of regulatory T cells (Tregs) and tolerogenic dendritic cells, which further suppress immune responses. The non-classical MHC class I molecule HLA-G expressed by MSCs contributes to this immunosuppressive effect. Furthermore, MSCs can disrupt the Th1/Th2 balance by increasing IL-4 and decreasing IFN-γ production, skewing the immune response towards a more anti-inflammatory Th2 phenotype. The lack of costimulatory molecules, low MHC class II expression, and secretion of immunosuppressive factors allow MSCs to evade immune recognition and actively create an environment that suppresses immune cell activation and proliferation [41,42].

Engineered mesenchymal stem cells (MSCs) are designed to improve their therapeutic efficacy by increasing their survival in vivo through various genetic modifications. The genetic modification of MSCs seeks to enhance their cellular processes, such as improving their ability to survive, migrate, home to target sites, and avoid senescence (premature aging) [17,43]. Overexpressing genes that promote cell survival and proliferation: Studies have shown that engineering MSCs to overexpress genes such as Akt, Bcl-2, and HIF-1α can increase their resistance to apoptosis (programmed cell death) and enhance their survival after transplantation [43]. Enhancing homing and adhesion to target sites: MSCs can be engineered to overexpress chemokine receptors such as CXCR4 or adhesion molecules such as integrins, which improves their ability to migrate and engraft at sites of injury or disease. Increasing resistance to oxidative stress and inflammation: Overexpressing antioxidant enzymes such as extracellular superoxide dismutase (ECSOD) can help MSCs better withstand the hostile inflammatory environment after transplantation [43]. Promoting angiogenesis and tissue regeneration: Engineering MSCs to secrete more growth factors such as VEGF, HGF, and IGF-1 can enhance their paracrine effects and stimulate blood vessel formation and tissue repair. These genetic modifications have been explored in various animal models of diseases such as cancer, cardiovascular disease, and radiation-induced injury and have shown improved therapeutic outcomes compared with unmodified MSCs. However, the results from human clinical trials have been mixed so far, and more research is still needed to optimize these engineered MSC therapies fully.

### 3.2. MSCs-Based Drug Delivery Strategies for Targeting of Tumor Cells

The development of drug delivery systems targeting MSCs for tumor tissues is a promising area of research with significant potential for clinical applications. MSCs have emerged as a valuable tool for delivering therapeutic agents to tumor sites. These systems aim to enhance the effectiveness and safety of drug delivery to tumors while minimizing toxicity to non-target tissues. Research in this field highlights several key points. MSC-based drug delivery systems have shown significant promise in delivering therapeutic agents to tumor sites [6,16]. Challenges include limited tumor tropism, broad biodistribution, and concerns about toxicity to non-target tissues [6]. Various synthetic engineering platforms are being developed to improve tumor-selective targeting and enhance clinical translation [6]. Strategies such as infecting MSCs with oncolytic viruses or activating prodrugs within the tumor microenvironment show promise in enhancing anti-tumor effects [6]. Overall, the development of drug-loaded MSCs as a targeted drug delivery system for tumor tissues holds great potential in improving the safety and efficacy of cancer therapies. Further research and optimization of these systems is essential to realize their full clinical benefits.

The therapeutic application of MSCs as drug-delivery vehicles for targeting tumor cells has shown promising results in cancer therapy. MSCs have been utilized as both a therapeutic agent and a carrier for anti-neoplastic agents, demonstrating the ability to alter the secretion pattern of cells in the tumor microenvironment, reduce angiogenesis, and inhibit metastasis [44,45]. Studies have highlighted that MSCs can effectively deliver chemotherapeutic drugs in a time- and concentration-dependent manner, leading to significant antineoplastic effects in various cancer cell lines [44]. Furthermore, drug-loaded MSCs have been found to reduce tumor size and inhibit angiogenesis in vivo studies, showcasing their potential as a targeted treatment for cancer [44,45]. The innate properties of MSCs, such as tumor tropism, self-renewal capacity, low immunogenicity, and lack of tumorigenicity, make them valuable candidates for drug delivery in cancer therapy [45]. Despite the need for improvements in aspects such as enhancing migration ability and regulating biodistribution to improve tumor delivery efficiency, MSC-based drug delivery systems hold promise for effective cancer-targeted therapy in the future [45]. Overall, utilizing MSCs as drug carriers for targeted delivery of therapeutic agents to tumor sites presents a novel and potentially impactful approach in the field of cancer treatment.

## 4. Stem Cell-Derived Extracellular Vesicle (EV) and Stem Cell Membrane-Coated Nanoparticles (SCMNPs)

**Figure 3:**
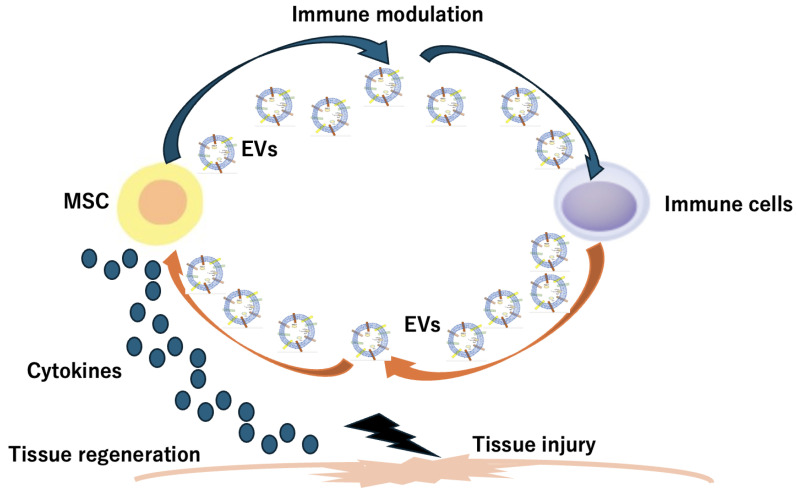
Stem cell-derived EVs are innovative approaches in targeted drug delivery and tissue repair. Stem cell-derived EVs play a role in intracellular communications for tissue regeneration.

SCMNPs and stem cell-derived EVs are innovative approaches in targeted drug delivery and tissue repair. Stem cell membranes can be modified to create membrane-coated nanoparticles, offering advantages such as strong targeting and low immunogenicity [46,47]. On the other hand, stem-cell-secreted EVs are natural nanoscale particles that transport biological molecules and facilitate cell-to-cell communication, showing promise in therapeutic applications because of their regenerative effects (Figure 3) [48,49]. These biomimetic nanocarriers derived from stem cells and EVs hold significant potential for targeted drug delivery systems and tissue repair therapies, offering safer and more controllable alternatives to traditional stem cell therapies [47,48]. 

Stem cell-secreted EVs play a crucial role in intercellular communication and have significant therapeutic potential. These vesicles, including exosomes and microvesicles, are rich in bioactive molecules such as proteins, lipids, nucleic acids, and organelles. They can transfer functional RNAs and proteins to target cells, influencing their functional status. Stem cell-derived EVs have been shown to guide chondrogenic differentiation, reduce adipogenesis, and modulate the functions of various cells both in vitro and in vivo [50,51]. Examples of stem cell-secreted EVs include exosomes and microvesicles, which are essential mediators in regenerative medicine and have emerged as promising tools for therapeutic applications. In addition, stem cell-derived EVs play a crucial role in regeneration by transferring bioactive cargo to recipient cells, modulating their activity, and promoting tissue repair. These EVs are submicron vesicles released from stem cells and contain various regulatory factors such as lipids, mRNA, and miRNA. They act as paracrine factors in cell-to-cell communication, regulating inflammation and promoting a shift from pro-inflammatory to pro-regenerative responses [14,51,52]. EVs have been shown to induce regenerative phenotypes, inhibit apoptosis, and modulate immune responses. They maintain stemness by delivering transcription factor mRNAs and effector molecules that regulate the proliferation, self-renewal, and differentiation of endogenous stem cells. Additionally, EVs can promote cell proliferation, neovascularization, and nerve regeneration in damaged tissues [14,51]. Overall, stem cell-derived EVs have low immunogenicity and strong therapeutic potential for tissue repair and regeneration. They are being explored as a promising alternative to cell-based therapies in regenerative medicine because of their ability to mediate immunomodulation, promote tissue repair, and contribute to the restoration of homeostasis [50,51].

The main methods for loading cargo into EVs can be divided into two broad categories: cell-based loading and non-cell-based loading. (i) Cell-based loading methods: Passive incubation: Small molecule drugs such as doxorubicin and curcumin can be loaded into EVs through simple incubation, though the loading efficiency is relatively low [53,54]. Transfection: Biomolecules such as small RNAs, mRNA, DNA, and proteins can be loaded into EVs through transfection. This method can achieve higher loading efficiency but requires transfection reagents, which may affect EV properties. (ii) Non-cell-based loading methods: Passive incubation: Similar to the cell-based approach, small molecule drugs can be loaded through simple incubation, but again with low efficiency [53,54]. Transfection: miRNAs and siRNAs can be loaded into isolated EVs through transfection, achieving enhanced efficiency but potentially altering EV structure and properties. Electroporation/Sonication/Freeze–thaw/Saponin: These methods can load a wider range of cargo, including small molecule drugs and biomolecules, into EVs, with relatively high efficiency. However, they may also increase EV instability and cause aggregation. In general, post-loading methods where cargo is loaded after EV isolation appear to be more controllable compared with pre-loading approaches. However, challenges remain in achieving high loading efficiency without compromising EV integrity and cargo functionality. A variety of pharmaceutical ingredients and biomolecular cargo have been successfully loaded into EVs using these different methods, including small molecule drugs, nucleic acids, and proteins [53,54,55,56].

On the other hand, SCMNPs have shown great promise in biomedical applications. Examples of these nanoparticles include erythrocyte-cancer hybrid membrane-coated nanoparticles designed for enhancing anti-tumor therapy efficacy [45], MSC membrane-coated nanoparticles used in antitumor and anti-inflammatory fields [57], and stem cell membrane-camouflaged superparamagnetic iron oxide (SPIO) nanoparticles employed for thermomagnetic therapy [58]. These examples highlight the diverse applications and potential of SCMNPs in targeted drug delivery and therapeutic interventions.

**Figure 4:**
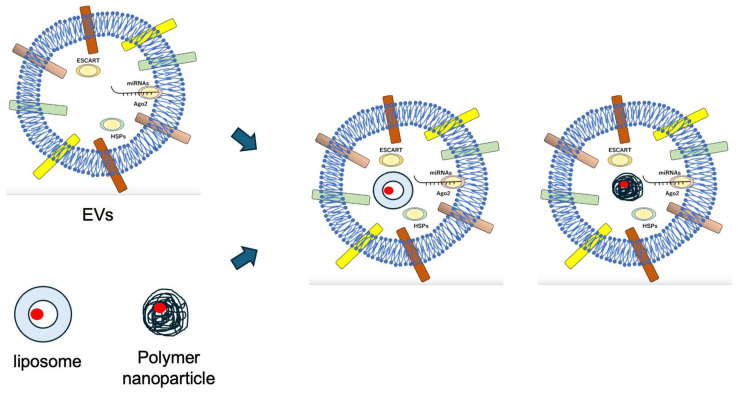
By combining synthetic nanoparticles with stem cell membranes, these nanomaterials exhibit enhanced targeted delivery and reduced immunogenicity.

The therapeutic applications of SCMNPs are significant in cancer therapy and drug delivery. SCMNPs offer advantages such as enhanced biocompatibility, strong targeting ability, immune evasion, and high drug-carrying capacity. They have been utilized to improve the stability, solubility, and half-life of drugs in tumor therapy. SCMNPs have emerged as a promising strategy to overcome the limitations of traditional nanomedicine, including low accumulation at target sites and rapid clearance from circulation. By combining synthetic nanoparticles with stem cell membranes, these nanomaterials exhibit enhanced targeted delivery and reduced immunogenicity, making them effective for treating various diseases such as cancer and inflammatory conditions [59,60,61] (Figure 4).

## 5. Stem Cell-Laden Scaffolds and Scaffold-Free Stem Cell Sheets

Stem cell-laden scaffolds play a crucial role in tissue engineering for regenerating complex tissues such as bone. These scaffolds are designed to support the growth and differentiation of stem cells, aiding in tissue repair and regeneration. By incorporating stem cells into scaffolds, researchers aim to mimic the natural environment of tissues, promoting cell survival, growth, and specific tissue formation. The use of stem cell-laden scaffolds has shown promising results in enhancing osteogenic differentiation, supporting long-term cell survival and growth, inducing neurogenetic differentiation of neural cells, and promoting vascularization and innervation in bone regeneration processes [58,62,63,64]. These innovative approaches hold great potential for advancing regenerative medicine by providing tailored environments for stem cells to develop into specialized tissues, offering new avenues for treating damaged or dysfunctional tissues effectively. The therapeutic applications of stem cell-laden scaffolds are significant in regenerative medicine. Stem cell-based therapeutics, when combined with scaffolds, offer enhanced potential for tissue repair and regeneration [65]. These scaffolds provide a 3D environment that supports stem cell survival, proliferation, and differentiation at the site of injury, overcoming challenges such as limited cell viability and retention. By mimicking the natural tissue microenvironment, these scaffolds play a crucial role in guiding stem cell fate decisions and controlling cell behavior to improve therapeutic efficacy [65,66,67]. 

The concept of scaffold-free stem cell sheets involves using cell sheets as a scaffold-free approach in tissue engineering. These sheets are created by culturing cells to form an intact sheet with their deposited extracellular matrix (ECM). Un traditional scaffold-based techniques, scaffold-free methods such as cell sheets do not rely on external structures for support, allowing for direct cell-cell and cell-matrix interactions [68,69]. This approach offers advantages such as faster, safer, and more reliable tissue construction compared with scaffold-based methods. Scaffold-free stem cell sheets have shown promise in enhancing bone regeneration, with studies demonstrating increased new bone formation and bone volume fraction when implanted into critical bone defects. Despite the potential benefits, challenges such as high production costs and time constraints for patient-specific cell sheet generation need to be addressed for wider acceptance and clinical translation [63,64,65,66,67,68,69,70,71,72]. Examples of scaffold-free stem cell sheets include cell sheets engineered using neurotrophic dental pulp stem/progenitor cells to promote and orient axon extension [72]. Another example is the use of scaffold-free hMSC tubes that enhance defect healing with delayed in vivo loading compared with loosely packed hMSC sheets [73]. Additionally, scaffold-free 3D cell sheet techniques have been developed to bridge the gap between 2D cell culture and animal models, showing promise in tissue repair and drug discovery [74]. These examples highlight the innovative applications of scaffold-free cell sheet engineering in regenerative medicine and tissue engineering.

The therapeutic applications of scaffold-free stem cell sheets encompass a promising approach in regenerative medicine. These cell sheets, which are engineered without the use of scaffolds, offer a unique method for tissue reconstruction and have shown significant potential in various clinical settings. By directly grafting cell-dense scaffold-free patches onto target tissue surfaces, these sheets facilitate local cell transplantation with enhanced cell retention and engraftment capabilities [71]. Scaffold-free stem cell sheets have been particularly beneficial in addressing clinical challenges associated with degenerative diseases and tissue repair. They have been utilized in diverse areas such as cornea, esophagus, heart, lung, middle ear, periodontal membrane, and cartilage regeneration, demonstrating safety and efficacy in promoting tissue repair and regeneration [71,75]. Additionally, advancements in cell sheet technology have enabled the development of bioactive scaffold-free cell sheets engineered from various stem/progenitor cells, such as MSCs, to promote axon extension and repair scarred myocardium after myocardial infarction [72,75].

Overall, scaffold-free stem cell sheets represent a cutting-edge approach in regenerative medicine, offering a versatile platform for tissue engineering and regeneration across different medical specialties. Their ability to enhance cell engraftment, promote tissue repair, and address clinical challenges associated with traditional cell therapies underscores their potential as a valuable tool in the field of regenerative medicine.

## 6. Therapeutic Applications in MSC-Based Drug Delivery

The current strategies and therapeutic application of MSC-based drug delivery focus on utilizing MSCs as a potential drug delivery system to enhance treatment efficacy and safety. Recent research highlights the promising potential of MSC-based drug delivery systems in treating various illnesses [76] (Table 1 and Table 2). However, challenges such as effectiveness, safety, and biodistribution need to be addressed for wider clinical applicability [22,77]. To overcome these challenges, cutting-edge technologies such as nanotechnology, genome engineering, and biomimetic technology are being developed to enhance MSC-based drug delivery systems. Studies emphasize the importance of understanding MSC biodistribution, pharmacokinetics, and pharmacodynamics for effective drug delivery. Additionally, the genetic engineering of MSCs is explored to improve safety and efficacy in cancer therapy [77,78]. The genetic engineering of MSCs is a promising approach to enhance their therapeutic efficacy in regenerative medicine and disease treatment [16,17,18,79]. The main goals of MSC genetic modification are to improve cellular survival, migration, homing, and adhesion to target sites, increase differentiation capacity and paracrine functions, and induce expression of specific proteins, growth factors, cytokines, enzymes, or microRNAs to achieve desired therapeutic effects. The genetic modification of MSCs can be achieved using viral vectors (e.g., lentiviruses, retroviruses) or non-viral methods (e.g., electroporation, nucleofection, lipid-based transfection) [18,79]. Viral vectors generally have higher efficiency but raise safety concerns, while non-viral methods are safer but less efficient [79]. Genetically engineered MSCs have been successfully used in various animal models to treat conditions such as myocardial infarction, tissue injury, ischemia, autoimmune diseases, radiation toxicity, and cancer [16,17]. For example, MSCs overexpressing antioxidant enzymes improved survival in radiation injury models. However, challenges remain in optimizing culture conditions, ensuring stable transgene expression, preventing cell death, and demonstrating safety and efficacy in clinical translation [79]. More research is needed to fully harness the potential of genetically modified MSCs as a therapeutic tool [17].

Clinical applications of MSC therapies have shown promising results in various diseases such as graft-versus-host disease, multiple sclerosis, Crohn’s disease, myocardial infarction, and acute respiratory distress syndrome [11,80,81,82]. Regulatory approvals for MSC products in different countries demonstrate the growing acceptance of MSC-based treatments. Despite advancements, challenges such as poor-quality control, stability issues, heterogeneity, and immunocompatibility need to be addressed for successful translation from research to clinical practice [83]. MSCs have shown great potential for clinical applications in regenerative medicine because of their ability to differentiate into various cell types, secrete bioactive factors, and modulate immune responses [82,84]. Over 300 clinical trials of MSC therapies have been completed, demonstrating a generally safe profile with tolerable side effects such as fever and pain at injection sites [82]. However, many early and late-stage clinical trials have failed, highlighting challenges in ensuring consistent quality, immunocompatibility, stability, and migratory capacity of MSCs. There are currently 10 approved MSC therapies worldwide for various indications, but none have yet been approved by the FDA in the United States [85]. Approved products include Cartistem for osteoarthritis (Korea), Alofisel for complex perianal fistulas in Crohn’s disease (Europe), and Ryoncil for steroid-refractory acute graft-versus-host disease in children (under FDA review) [84,85]. MSCs have been studied in over 160 clinical trials for neurological disorders such as Alzheimer’s, Parkinson’s, multiple sclerosis, and amyotrophic lateral sclerosis [11]. While preclinical studies in animal models showed promise, results from small human trials have been mixed, with some indications of safety and moderate clinical benefits. MSCs are also being investigated for regenerative applications in the heart, bone, cartilage, skin, and other tissues [81,82]. Intravenous injection appears to be the most common administration route. While MSC therapies hold great promise, significant challenges remain in ensuring consistent product quality and demonstrating clear efficacy in clinical trials. Ongoing research aims to optimize MSC isolation, expansion, and delivery to improve therapeutic outcomes.

**Table 1:** Therapeutic applications in MSC-derived EVs.

MSC Origin	Target Therapy	Registration Year	Drug/Biomolecule Used	Models	Potential Outcomes	References
Cord blood	Type I Diabetes Mellitus (T1DM)	2014		streptozotocin-induced diabetic rats	reducing blood glucose levels, improving insulin sensitivity, and inhibiting β-cell apoptosis	[86,87]
Umbilical cord	Chronic kidney injury	2016	lipopolysaccharide	models of acute kidney injury and chronic kidney disease	reducing oxidative stress, inflammation, and fibrosis.	[88,89,90]
Human umbilical cord	Healing of Macular holes	2017		macular degeneration animal models	alleviation of inflammation and damage	[91,92]
Bone marrow	Bronchopulmonary Dysplasia	2019		experimental models of bronchopulmonary dysplasia	reducing inflammation, improving alveolarization, and promoting angiogenesis	[93,94,95,96]
Bone marrow	Dystrophic Epidermolysis Bullosa	2020		Dystrophic Epidermolysis Bullosa mouse models	promoting wound healing, reducing blister formation, and improving type VII collagen expression	[51,77,97,98]
Adipose tissue	Human Osteochondral Explants	2020		in vitro and ex vivo models, including human osteochondral explants	bone and cartilage regeneration, as well as in attenuating osteoarthritis progression	[99,100,101,102,103]
Bone marrow	A Tolerance Clinical Study on Aerosol Inhalation	2020		preclinical models	regenerative and anti-inflammatory properties	[77,104,105]
Adipose Tissue	Severe Novel Coronavirus Pneumonia	2020		porcine model	reducing virus entry and lung inflammation, anti-inflammatory, immunomodulatory, and regenerative properties	[51,106,107,108]
Adipose Tissue	ARS-CoV-2 Associated PneumoniaSARS-Cov2 pneumonia	2020		SARS-CoV-2 pneumonia and ARDS models	reducing lung inflammation, promoting epithelial and endothelial recovery, and enhancing alveolar fluid clearance	[109,110,111]
Human umbilical cord	Dry Eye in Patients With cGVHD	2020		animal models	promoting corneal epithelial wound healing by modulating inflammation, promoting angiogenesis, and stimulating stem/progenitor cell proliferation	[112]
Adipose tissue	Periodontitis	2020		pre-clinical animal models	alleviating oxidative stress, inhibiting inflammation, and promoting tissue regeneration	[113,114,115,116]
Wharton’s jelly	Chronic Ulcer Wounds	2020		preclinical models	accelerating wound healing	[77,117]
Bone marrow	Multiple Organ Dysfunction Syndrome (MODS) After Surgical Repair of Acute Type A Aortic Dissection	2020		ischemia-reperfusion injuries	improving organ function (liver, lung, coagulation) and reducing MODS severity	[92]
Adipose tissue	Pulmonary Infection	2020	silica	mouse model of silica-induced lung inflammation and fibrosis	reducing collagen fiber content, granuloma size, and the number of macrophages and decreasing the expression levels of pro-inflammatory cytokines IL-1β and TGF-β in the lungs.	[118]
Adipose tissue	Alzheimer’s Disease (AD)	2020		in vitro and in vivo models of AD	ameliorating AD pathology and neuronal apoptosis	[104,119,120]

**Table 2:** MSC-derived EVs for cancer therapy.

Target Therapy	MSC Origin	Cargo Type	Cargo	Outcome	Reference
Breast cancer	Bone marrow	miRNA	miR-100	Suppressed angiogenesis	[121]
	Bone marrow		miR-23b	Promoted dormancy	[122]
	Bone marrow		miR-16	Suppressed angiogenesis	[123]
Glioma	Bone marrow		miR-124/miR-145	Decreased migration and self-renewal	[124]
	Bone marrow		miR-146b	Inhibited tumor growth	[125]
	Bone marrow		miR-124a	Reduced viability	[121]
	Bone marrow		miR-133b	Inhibited proliferation, invasion, and migration	[126]
Osteosarcoma	Bone marrow		miR-143	Suppressed migration	[127]
Hepatocellular carcinoma	Adipose		miR-122	Growth inhibition	[128]
Prostate cancer	Adipose		miR-145	Suppressed cancer progression	[129]
Multiple myeloma	Bone marrow		miR-15a	Growth inhibition	[130]
Pancreatic cancer	Bone marrow		miR-1231	Inhibited cancer activity	[131]
	Bone marrow	siRNA	siKrasG12D-1	Induced apoptosis	[132]
Hepatocellular carcinoma	Bone marrow		siGRP78	Growth inhibition	[133]

## 7. Conclusions

MSCs have emerged as a promising platform for targeted drug delivery because of their unique properties. MSCs have an innate ability to migrate and home to sites of inflammation, including tumors, which can be exploited to deliver a variety of therapeutic agents directly to the target site. MSCs can be readily isolated from various tissues, such as bone marrow, adipose tissue, and umbilical cord, and can be expanded ex vivo while maintaining their phenotype and multipotent differentiation potential. MSCs have low expression of major histocompatibility complex (MHC) molecules, allowing them to evade detection and clearance by the host immune system, which is advantageous for allogeneic cell-based therapies. The field still faces challenges, such as limited tumor tropism, broad biodistribution, and the need for improved quantification and standardization of MSC-based drug delivery approaches to facilitate effective clinical translation.

MSCs have several key properties that make them competent biomedical candidates for regenerative therapies. MSCs can alleviate abnormal immune responses, which is beneficial for tissue repair. MSCs can generate growth factors that stimulate the repair process. MSCs can differentiate into various cell types, including osteoblasts, chondrocytes, myocytes, and adipocytes, allowing them to regenerate different tissues. Induction factors and scaffolds are critical for enhancing the therapeutic effects of MSCs. Induction factors accelerate the repair process, while scaffolds provide the environment for MSC proliferation, differentiation, and mechanical stimulation. Overall, the unique properties of MSCs, combined with the use of appropriate induction factors and scaffolds, make them a promising cell source for regenerative medicine and tissue engineering applications.
